# Notes on Certain Vegetable Poisons

**Published:** 1857-04

**Authors:** William Pickells

**Affiliations:** Cork.


### NOTES ON CERTAIN VEGETABLE POISONS.

By WILLIAM PICKELLS, M.D., Cork.

(*Continued from* vol. ii, p. 382.)

Hemlock. Dr. Osborne seeks to prove, in answer to some remarks of Dr. Christison, that the State poison (κωνειον) of the Athenians, used in the case of Socrates, was essentially hemlock. The question of the poison used in the case of Socrates, having been so long and so much discussed, may be supposed to have become trite; it appears, however, still to possess an interest, relating, as it does, to the martyrdom of the "prince of philosophers". Dr. Christison had said, in an article published in *The Transactions of the Royal Society of Edinburgh*, that

"the State poison of the Athenians must be considered as producing spasm and coldness of the extremities, gradually advancing to the internal parts; causing death eventually by acting either on the heart or respiration, without affecting the functions of the mind to the very last—a combination of symptoms not to be produced by any poison now known!"

Dr. Osborne meets the difficulty chiefly by a comparative review of the effects of hemlock, according to modern observations, and of the series of symptoms which preceded its fatal effects in the case of Socrates; inferring, "that the resemblance is so close, that no reasonable doubt can be entertained of their being results of the same agency." The last words of Socrates, spoken in the request to Crito, were, he infers, "spoken under the influence of a slight delirium, produced by the hemlock, and to be considered as the results of a narcotic poison." He cites cases to shew that the delirium produced by hemlock is "of a peculiar character, slight—so very slight, in some cases—as to be scarcely sufficient to attract notice."

There is a remarkable passage in Galen, which, as it has occurred to me, may affect the question. "It is to be regretted," he says, "that Plato has not left an answer to the objection to the immortality of the soul, drawn from the delirium of fever, and that produced by the cicuta"; thus instituting an analogy between the two. The objection to the immortality of the soul is, it will be perceived, a mere cavil, a disingenuous carping or play on words (delirium implying alienation of mind), and, if coming from Galen, unworthy of him, whose admirable essays on the *Use of the Parts of the Human Body*, as proving design, have been each in its order appropriately entitled "Hymn to the Divinity!" The passage appears, however, to be available, so far as regards the present question. "No doubt can be entertained," Dr. Osborne says, "of the identity of κωνειον and cicuta. Now the delirium of fever, we know, is commonly not so very slight as not to be well marked; it being often even severe to a degree, rarely, however, furious or maniacal. The last words spoken by Socrates might have been, as we would hope they were, pronounced under the influence of a slight delirium; but that the delirium usually produced by the cicuta was not of a peculiar, very slight, or scarcely noticeable character, is evident from the analogy instituted between it and the delirium of fever, and upon the one species of delirium, as upon the other, a cavil, however captious and frivolous, being raised against the immortality of the soul. Galen derives κωνειον from κωνεω, volvo, whence κωνος, a top; vertigo, it would appear, being a common effect of it.

"A number of writers," Dr. Osborne says, "have been led, one after another, to set down furious delirium as a symptom of poisoning with hemlock. The foundation of this statement rests," he observes, "on the authority of two cases, both unworthy of credit. The first, said to be related by Kircher, given in Wæpfer's treatise *De Cicutâ Aquaticâ* (cicuta virosa), is of two monks, who, after having partaken of a dish composed of the roots of hemlock boiled up with meat, became almost immediately deranged; the one, rushing into a neighbouring lake, called out that he had become a goose; the other ran into the streets, shouting that he was a duck, and required the river to extinguish the fire in his intestines." The story (though there are instances of hallucination or monomania as strange) should certainly be received with suspicion. Supposing it true, however, that the effects were produced by a vegetable poison, it is not sufficiently authenticated that they were produced by hemlock.

In a paper published by me, some years since, in vol. lxvii of the *Edinburgh Medical and Surgical Journal*, on the poisonous effects of the œnanthe crocata, as it occurs in the south of Ireland, a case is given of furious delirium produced by that species of hemlock. "A lad, after having eaten of the root of the plant, mistaking it for celery, ran stark mad, but came to his senses again next morning." The case was given on the authority of "many eye witnesses." In another case, mentioned in same paper, "a woman, living in the town of Dungarvan, having brought home a root of the plant for a poultice, one of the children ate of it, by mistake for a parsnip, and was seized in consequence with risus sardonicus and other symptoms, but recovered by the administration of melted butter."*

The second narrative rejected by Dr. Osborne, as unworthy of credit, is by Matthiolus, of a vine-dresser and his wife, who took hemlock roots in mistake for parsnips. "When they awoke in the night, they became quite furious, wandering about in the dark, and knocking their heads against the walls, so that their faces in the morning were swollen with the effused blood." "It was impossible," it is stated, "for Matthiolus to identify the plant, as the leaves were only beginning to shoot." This

* In mentioning the *Edinburgh Medical and Surgical Journal*, I take the opportunity of correcting an inaccuracy into which I find I fell, in a paper "On Hæmorrhagic Diathesis," published by me in xol. lxxvii of that Journal. In a foot note, p. 6, alluding to certain very bold but ill supported hypotheses, it is said that, to some, the authors might seem to merit the sarcasm applied by Reid, in his Essays, to certain theorists, as in their neomania, or love of paradox, "hunting after bladders of soap in the land of chimera." It should have been, by Helvetius, on Man.

narrative I would not reject as unworthy of credit; but, as the plant could not be identified, would attribute what took place, rather to their having eaten the roots of hyoscyamus, than those of hemlock.

In the paper of mine, already referred to, an instance well authenticated is given, of "a whole family, living at Lambay Island, near Dublin, having been driven mad, attempting to bite everything which came in their way, in consequence of having eaten the roots of hyoscyamus, which had been brought home and boiled as parsnips." "The insane root" of Shakespeare, "that taketh the reason prisoner", is supposed to be the hyoscyamus.

Whatever difficulty may exist, and difficulty, I fear, will still be, in reconciling the physiological action of hemlock on the mind with the account of Plato, it appears, however, pretty certain that hemlock of one species or of the other was, if not the sole, the essential ingredient in the State poison, or *κωνειον*, of the Athenians; which, used in the cases of Socrates and of other most illustrious men, has brought on the Athenian name, and on popular liberty itself, the stain "of decreeing to the same citizen, the hemlock on one day, and statues on the next." Xenophon, towards the conclusion of the *Memorables*, in reflecting on the death of Socrates, observes: "In my opinion, he met a fate acceptable to the gods; for he left after him the most disagreeable part of life,* and met the easiest of deaths," "*τῶν θανάτων τοῦ ῥᾴστου ἔτυχεν.*" Hence it might appear, that death by the *κωνειον* was reputed to be the easiest of deaths.†

Condemnation to drink hemlock, as a mode of capital punishment, was not peculiar to the Athenians. It was practised in Massilia, the present Marseilles; a quantity of the expressed juice of the plant being, as at Athens, kept at the public cost, ready to be brewed on occasion. A paragraph has been lately going the round of the newspapers, apparently copied from a Marseilles newspaper, or other source, headed "Curious Law" (curious, indeed, if true). "There was kept in former times, in our city of Marseilles, a poison prepared from hemlock at the public charge, for those who had a mind to hasten their end; they having first, before the Senate, given an account of the reason and motives of their design. It was not otherwise lawful for the citizens to do violence to themselves." Suicide

---

* He was seventy or eighty years of age.

† The real opinions of Socrates are to be studied rather in Xenophon than in Plato.

was permitted, in certain cases, by some of the ancient philosophers. There were states in which it was even permitted by law.*

In modern history, Eric XIV of Sweden, son of Gustavus Vasa, was put to death by being made to drink hemlock. He had been dethroned by his subjects for cruelty and treachery; and, after an imprisonment of eight years at Gripsholm, "took," it is stated, "a draught of hemlock in 1577, by command of his brother, King John." The species of hemlock used in this case, was doubtless the cicuta virosa; this plant, which appears to prefer a northern or cold habitat, being frequent in the far north of Europe, and also in Siberia.

Wæpfer, in his treatise on the cicuta virosa, above referred to, states that it is poisonous to swine. The professor of agriculture to Queen's College, in this city (Mr. Murphy), informs me that the proprietor of a farm on the river side, near this city, told him that he some time since lost a number of pigs, in consequence of their having eaten of the *œnanthe crocata*, which had been cast ashore in considerable quantities after a flood. The poisonous effects of these species of hemlock on swine are the more remarkable, as in America, swine, it is said, eat the rattle-snakes with impunity; farmers keeping pigs on their lands for the purpose of clearing them of rattle-snakes!

---

### ON THE DISEASES OF COLLIERY OPERATIVES; AND THEIR PREVENTIBLE CAUSES.

* Marseilles was founded by the Ionians. Among the constitutions of various states, to which Aristotle refers in his work on Government, is that of Marseilles.

